# Clinical Management of a Rare Hereditary Bleeding Disorder in an Adult: Glanzmann Thrombasthenia

**DOI:** 10.7759/cureus.72391

**Published:** 2024-10-25

**Authors:** Reya Mecca, N. Senthil, Suja Lakshmanan, Archa Anna Anil

**Affiliations:** 1 Internal Medicine, Sri Ramachandra Institute of Higher Education and Research, Chennai, IND

**Keywords:** autosomal recessive inheritance, deficient platelet aggregation, gingival hemorrhage, hereditary hemorrhagic thrombasthesia, mucocutaneous bleeding, platelet abnormality

## Abstract

Glanzmann thrombasthenia (GT) is a rare autosomal recessive bleeding disorder affecting the megakaryocyte lineage and is associated mainly with mucocutaneous bleeding. We herein report a woman in her early 40s, with no known comorbidities, who presented with severe gingival bleeding and severe fatigue. Past history revealed recurrent gingival bleeding, bruising, and heavy menstrual bleeding. Platelet aggregation studies revealed normal aggregation. Finally, a diagnosis of GT with microcytic hypochromic anemia was established after ruling out all the other differential diagnoses. The patient was transfused with multiple units of packed cells (PCs), random donor platelets (RDPs), and single donor platelets (SDPs) and treated with intravenous (IV) and topical tranexamic acid. Our patient presented with a rare bleeding disorder very late in life. GT can be potentially life-threatening and is difficult to diagnose in the early stages. Therefore, it is imperative to highlight the importance of early diagnosis of this rare condition to provide supportive care for a good prognosis.

## Introduction

It was first documented by Dr. Eduard Glanzmann in 1918 as “hereditary hemorrhagic thrombasthenia,” where he described the bleeding disorder as a novel platelet abnormality with flawed clot retraction and an abnormal appearance on stained film [1]. The description has since evolved as Glanzmann thrombasthenia (GT) being characterized as an autosomal recessive genetic disorder with normal to subnormal platelet levels, prolonged bleeding time, and deficient platelet agglutination [1-3]. This abnormality ultimately manifests as mucocutaneous hemorrhage of varying intensities that can most often be highly morbid, requiring multiple transfusions. The diagnostic features of GT, including the absence of platelet aggregation, were established only in 1964 via a report of 15 French patients by Caen et al. [4]. Though GT is an extremely rare entity globally, it is relatively common in consanguineous communities [5].

We present a case of a female in her early 40s with features of a bleeding disorder in the form of gingival bleeding and bruising leading to severe microcytic hypochromic anemia. She was extensively evaluated for her symptoms and finally was diagnosed with GT, as evidenced by normal platelet aggregation with ristocetin. She was then transfused multiple times with packed cells (PCs), random donor platelets (RDPs), and single donor platelets (SDPs), followed by intravenous (IV) tranexamic acid, showing a significant clinical improvement. We herein report this case due to its rarity in the presentation.

## Case presentation

A South Asian female in her early 40s, born to non-consanguineous parents, was admitted with a two-week history of continuous gum bleeding accompanied by severe fatigue and dizziness. She had initially been treated at a primary care facility, where she received five units of packed red cells due to extremely low hemoglobin (Hb) (4.0 g/dL) before being referred to our tertiary care center for further evaluation.

Further history revealed recurrent spontaneous bruising and prolonged gingival bleeding during tooth brushing since childhood, though these symptoms had never been properly assessed. She denied any recent trauma, joint bleeding, bleeding from other orifices, or gastrointestinal bleeding. However, she had undergone a hysterectomy 10 years earlier for heavy menstrual bleeding, suspected to be due to endometriosis, and also reported multiple past blood transfusions. There was no history of medication use or significant family history of bleeding disorders up to the third generation.

On examination, she was afebrile with stable vitals. Severe conjunctival pallor was noted. Oral examination revealed spontaneous gingival bleeding, causing mild staining, though the attached gingiva was firm and resilient. Multiple ecchymotic patches were observed on both lower limbs. Systemic examination was unremarkable, with no palpable splenomegaly and a normal liver.

Routine laboratory investigations, including blood glucose, glycated Hb (HbA1c), serum electrolytes, renal profile, and liver parameters, were within normal limits. Hb was reduced to 8.5 g/dL (12-15 g/dL), with platelet levels at 137 × 10^9^/L (150-400 × 10^9^/L). A peripheral smear showed a low red blood cell (RBC) count, with microcytic and hypochromic RBCs, along with anisocytosis and tear drop cells, indicating microcytic hypochromic anemia. White blood cells were unremarkable in terms of number, morphology, and distribution, with adequate platelet levels.

Iron studies revealed low serum iron levels with an increased total iron-binding capacity. Bleeding time and clotting time were within normal limits. Prothrombin time (PT) was 12.0 seconds (11-16 seconds), activated partial prothrombin time (aPTT) was 26.8 seconds (26-40 seconds), and the international normalized ratio (INR) was normal. Thrombin time was 18.1 seconds (16.4-18.9 seconds), and serum fibrinogen levels were 205 mg/dL (250-520 mg/dL) (Table 1). A clot retraction test showed no clot formation even after 24 hours, suggesting GT.

**Table 1:** Significant initial laboratory investigations to affirm a bleeding disorder

S. no.	Investigation	Result	Biological reference interval	Units
1.	Hemoglobin (Hb)	8.5	12-17 (male); 12-15 (female)	g/dL
2.	Total leukocyte count (TLC)	5500	4,000-11,000	cmm
3.	Platelet count	137	150-400	×10^9^/L
4.	Prothrombin time (PT)	12.0 control-12.2	11-16 seconds	-
5.	Activated partial prothrombin time (aPTT)	26.8 control-25.4	26-40 seconds	-
6.	International normalized ratio (INR)	0.97	-	-
7.	Serum iron	37.58	60-180	mcg/dL
8.	Total iron binding capacity	434.10	240-400	mcg/dL
9.	Bleeding time	Two minutes 30 seconds	2-7	minutes
10.	Clotting time	Five minutes 30 seconds	3-10	minutes
11.	Thrombin time	18.1	16.4-18.9	seconds
12.	Serum fibrinogen	205	250-520	mg/dL
13.	Serum von Willebrand factor (VWF)	148.9	47.8-173.2	IU/dL
14.	Viral markers: hepatitis B surface antigen (HBsAg), hepatitis C virus (HCV), HIV-I and II	Negative	-	-

Further tests revealed normal levels of serum von Willebrand factor (vWF) and factor VIII, along with a negative factor XIII screen. Additionally, viral markers were non-reactive, and antinuclear antibodies were negative.

On the basis of history, examination and laboratory investigations, von Willebrand disease, platelet functional defect, Bernard-Soulier syndrome, GT, idiopathic thrombocytopenic purpura (ITP), and clotting factor deficiency (hemophilia) were identified as possibilities. 

To confirm the diagnosis platelet aggregation studies were performed. Platelet aggregation was induced with adenosine diphosphate, epinephrine, arachidonic acid, and ristocetin. In our patient, platelet aggregation was only seen with ristocetin, confirming the diagnosis of GT (Table 2). Normal serum vWF levels ruled out von Willebrand disease, a normal platelet count reduced the likelihood of ITP, and normal factor VIII levels excluded hemophilia. Finally, based on the clinical course of recurrent gingival bleeding and spontaneous bruising with a history of heavy menstrual bleeding supported by laboratory investigations, the patient was diagnosed with GT. 

**Table 2:** Platelet aggregometry results indicating platelet aggregation with ristocetin

Agonist	Adenosine diphosphate (mmol/L)		Epinephrine (mmol/L)		Arachidonic acid (mmol/L)		Ristocetin (mg/mL)		
Percentage aggregation	2	5	2	40	1	8	1.2	0.6	2
	1.0	-	0.0	-	2.9	-	58.0	-	-

After diagnostic confirmation of GT followed by a dental and hematology opinion, she was started on IV tranexamic acid 500 mg once daily, followed by topical tranexamic acid powder and topical metronidazole with little improvement. However, her symptoms intensified. She was then transfused with one unit of SDP. Hb was then recorded as 6.2 g/dL (12-15 g/dL). In view of severe bleeding and a significant drop in Hb, she was then transfused with one unit of PC while continuing regular IV tranexamic acid and topical tranexamic acid powder. Though dosages were increased to the maximum, she did not improve clinically, and bleeding continued. Another four units of RDPs were transfused owing to the continuously dropping platelet levels, ceasing the gingival bleeding. Hb following transfusion was noted as 8.8 g/dL (12-15 g/dL) with a platelet level of 115 × 10^9^/L (150-400 × 10^9^/L).

The patient had an uneventful recovery and was advised to continue oral tranexamic acid 500 mg. She is currently well after six months of recovery with occasional mild gingival bleeding on vigorous brushing of teeth, and Hb recorded as 8.5 g/dL. She has been advised to switch to a soft bristle toothbrush and careful oral hygiene with regular follow-up visits at the dental outpatient. 

## Discussion

Platelets represent a central component of many restorative physiological processes, including hemostasis, and hence, any disruption in platelet function, whether acquired or inherited, is bound to generate bleeding. Acquired platelet disorders are more commonly encountered during regular clinical practice compared to their inherited counterpart, which are quite rare and, until recently, have been under less scrutiny [6]. In the recently reported literature, GT has gained enough recognition to be described as one of the first disorders to define the role of GPIIb/IIIa in the hemostasis cascade while also serving as a template for key research findings in hematology [1,3,6].

GT is a rare inherited bleeding disorder with a relatively high incidence in consanguineous populations like Iraqi Jews, French gypsies, Jordanian nomadic tribes, and South Indian Hindus, whereas GT is extremely rare in other parts of the world. Hence, it becomes difficult to estimate the worldwide prevalence accurately [3,7]. In a recent report from Pakistan, consanguinity concerning GT was reported as 85.3% and 63% in the Indian population [8,9]. Furthermore, Nair et al. reported GT in 42 families, with all of them demonstrating a strong predilection to consanguinity, unlike in our case, where no connection to consanguinity was reported [10]. However, Lowe et al. described the worldwide prevalence of GT as one in one million [11]. Most often, severe symptoms of GT are found at a median age of nine years two months and a range of eight months to 26 years, as opposed to our patient, who presented with severe bleeding much later in life [8].

Over time, the pathophysiology behind GT has come to be well understood. Glycoprotein IIb/IIIa (GPIIb/IIIa) is a transmembrane receptor on the surface of platelets that mediates the binding of adhesive proteins like fibrinogen, vWF, fibronectin, and vitronectin [12]. GT is characterized by a qualitative and/or quantitative defect in the platelet surface receptor GPIIb/IIIa, leading to impaired platelet aggregation and diminished clot retraction. GT has further been classified into three subtypes, with type I being defined as a total absence of the GPIIb/IIIa complex. In type II, only a partial deficiency of the above-mentioned protein is noted, whereas in the variant type, GPIIb/IIIa are functionally impaired [13,14]. Such individuals then present with a life-long risk of bleeding ranging from mild to severe, even life-threatening hemorrhage [15]. Patients are usually expected to present with easy bruising, epistaxis, gingival bleeding, and prolonged bleeding following minor trauma such as dental extractions [16]. 

GT can be diagnosed clinically and strongly supplemented by laboratory evidence. Normal platelet morphology with a normal platelet count are characteristics of GT. Additionally, prolonged bleeding time, absent or decreased clot retraction, and normal platelet aggregation in the presence of ristocetin are the most common findings in GT, as seen in our case. Platelet aggregation test is the gold standard for diagnosis of GT as aggregation would be absent with epinephrine, collagen, arachidonic acid, and ADP due to the dependence of these factors on fibrinogen attachment to the platelet for aggregation as opposed to normal response in the presence of ristocetin owing to its independence from fibrinogen [2,17]. Flow cytometric analysis using monoclonal antibodies can be used to provide a more definitive diagnosis, yet its availability and affordability in developing countries like ours are major limiting factors [12].

According to the available literature, there is no definitive treatment for GT. The overall morbidity and mortality have been difficult to estimate given its rarity, but in the given studies, hemorrhage is the main concern, and supportive care remains critical [3,7,18]. Platelet transfusion has been described as the first line of treatment before any invasive procedure or after any heavy bleeding episode [19]. However, Harrison et al. argue the risk of alloimmunization that comes along with platelet transfusions, and it must remain the last resort as a treatment modality only reserved in cases of very severe bleeding [20]. Management of GT mainly aims at preventing bleeding episodes by educating patients and their family members to avoid any possible trauma and improving oral hygiene while carefully avoiding anti-platelet drugs. In addition, the current standard of care for mild to moderate bleeding episodes employs the use of local measures alone or in conjunction with anti-fibrinolytic therapy [15]. It is also important to note that Mahmood et al. treated 16.8% of their cohort of patients with fresh frozen plasma and cryoprecipitate, which remains unjustified currently, exposing patients to possible complications [8]. This opens up a need for further randomized controlled trials to standardize a treatment protocol that clarifies the benefits and possible harmful effects of various available treatment modalities. 

## Conclusions

GT is a rare bleeding disorder due to defective GPIIb/IIIa proteins ultimately leading to deficient platelet aggregation. As per reports, this disorder is most commonly found in consanguineous populations and rarely in other parts of the world. Patients characteristically present with easy bruising, orifical bleeding, gingival bleeding, and prolonged bleeding after minor traumas. Platelet aggregation studies, along with flow cytometric analysis, are the gold standard tests that confirm the diagnosis of GT, followed by supportive treatment personalized to the symptoms of the patient.

GT is an uncommon bleeding disorder with varying manifestations, but it can be absolutely fatal without proper supportive care. Increased awareness among physicians can help in prompt diagnosis and then the development of effective management strategies and, ultimately, sustainable use of medical resources.
